# Evaluation of Met-Val-Lys as a Renal Brush Border Enzyme-Cleavable Linker to Reduce Kidney Uptake of ^68^Ga-Labeled DOTA-Conjugated Peptides and Peptidomimetics

**DOI:** 10.3390/molecules25173854

**Published:** 2020-08-25

**Authors:** Shreya Bendre, Zhengxing Zhang, Hsiou-Ting Kuo, Julie Rousseau, Chengcheng Zhang, Helen Merkens, Áron Roxin, François Bénard, Kuo-Shyan Lin

**Affiliations:** 1 Department of Molecular Oncology, BC Cancer, Vancouver, BC V5Z 1L3, Canada; sbendre@bccrc.ca (S.B.); zzhang@bccrc.ca (Z.Z.); hkuo@bccrc.ca (H.-T.K.); jrousseau@bccrc.ca (J.R.); cczhang@bccrc.ca (C.Z.); hmerkens@bccrc.ca (H.M.); aroxin@bccrc.ca (Á.R.); fbenard@bccrc.ca (F.B.); 2 Department of Functional Imaging, BC Cancer, Vancouver, BC V5Z 4E6, Canada; 3 Department of Radiology, University of British Columbia, Vancouver, BC V5Z 1M9, Canada

**Keywords:** radiopharmaceuticals, kidney uptake, cleavable linkers, neutral endopeptidase (NEP), renal brush border enzymes, prostate-specific membrane antigen (PSMA), cancer imaging and therapy

## Abstract

High kidney uptake is a common feature of peptide-based radiopharmaceuticals, leading to reduced detection sensitivity for lesions adjacent to kidneys and lower maximum tolerated therapeutic dose. In this study, we evaluated if the Met-Val-Lys (MVK) linker could be used to lower kidney uptake of ^68^Ga-labeled DOTA-conjugated peptides and peptidomimetics. A model compound, [^68^Ga]Ga-DOTA-AmBz-MVK(Ac)-OH (AmBz: aminomethylbenzoyl), and its derivative, [^68^Ga]Ga-DOTA-AmBz-MVK(HTK01166)-OH, coupled with the PSMA (prostate-specific membrane antigen)-targeting motif of the previously reported HTK01166 were synthesized and evaluated to determine if they could be recognized and cleaved by the renal brush border enzymes. Additionally, positron emission tomography (PET) imaging, ex vivo biodistribution and in vivo stability studies were conducted in mice to evaluate their pharmacokinetics. [^68^Ga]Ga-DOTA-AmBz-MVK(Ac)-OH was effectively cleaved specifically by neutral endopeptidase (NEP) of renal brush border enzymes at the Met-Val amide bond, and the radio-metabolite [^68^Ga]Ga-DOTA-AmBz-Met-OH was rapidly excreted via the renal pathway with minimal kidney retention. [^68^Ga]Ga-DOTA-AmBz-MVK(HTK01166)-OH retained its PSMA-targeting capability and was also cleaved by NEP, although less effectively when compared to [^68^Ga]Ga-DOTA-AmBz-MVK(Ac)-OH. The kidney uptake of [^68^Ga]Ga-DOTA-AmBz-MVK(HTK01166)-OH was 30% less compared to that of [^68^Ga]Ga-HTK01166. Our data demonstrated that derivatives of [^68^Ga]Ga-DOTA-AmBz-MVK-OH can be cleaved specifically by NEP, and therefore, MVK can be a promising cleavable linker for use to reduce kidney uptake of radiolabeled DOTA-conjugated peptides and peptidomimetics.

## 1. Introduction

The use of low molecular weight radiolabeled peptides and antibody fragments for applications in oncology is rapidly gaining momentum [1,2,3,4,5]. Such a site-directed radiation delivery involves targeting of certain specific receptors overexpressed on the surface of cancer cells for the purpose of targeted imaging and radionuclide therapy [6]. While these oncophilic molecules serve as biological targeting vectors, they commonly exhibit very high and sustained renal uptake [7]. This is caused by either a high renal expression of cancer markers targeted by these oncophilic molecules, megalin-cubilin mediated endocytosis and transcellular transport, or lysosomal proteolysis following glomerular filtration and renal reabsorption [7,8,9]. This reduces detection sensitivity for lesions adjacent to kidneys and lowers the maximum tolerated dose for radiotherapy.

Arano et al. reported an effective strategy to reduce kidney uptake of radiopharmaceuticals by incorporating specific cleavable linkages into them, with the Met-Val-Lys (MVK) sequence found to be the most effective thus far [1]. Use of this strategy is attributable to recognition and cleavage of MVK between Met-Val residues by a metalloendopeptidase enzyme called neutral endopeptidase or neprilysin (NEP) [10]. This enzyme is found in abundance on the renal brush border membrane lining, particularly of the proximal convoluted tubules [11]. Arano et al. exploited this renal brush border enzyme and designed a NOTA(1,4,7-triazacyclononane-1,4,7-triacetic acid)-conjugated ^67/68^Ga-labeled antibody fragment bearing this MVK linker sequence to successfully lower renal uptake by 80% at 3 h post-injection (p.i.) without loss of tumor uptake [12].

By adopting this design strategy Zhang et al. recently reported the comparison of two radiolabeled Exendin 4 derivatives for imaging the expression of the glucagon-like peptide-1 receptor (GLP-1R) with positron emission tomography (PET) [13]. GLP-1R is highly expressed in insulinomas. However, the very high and sustained uptake of radiolabeled GLP-1R-targeting Exendin 4 derivatives in kidneys hinders the application of these tracers for detecting insulinomas. Compared with [^68^Ga]Ga-NOTA-Cys^40^-Leu^14^-Exendin 4, the derivative bearing the MVK linker ([^68^Ga]Ga-NOTA-MVK-Cys^40^-Leu^14^-Exendin 4) had similar tumor uptake values but only one third of kidney uptake at 2 h p.i., greatly enhancing the tumor-to-kidney contrast and detection sensitivity [13].

Despite successful application of this strategy to lower kidney uptake of radiolabeled peptides, the use of NOTA as a radiometal chelator unfortunately excludes application for using therapeutic isotopes like ^177^Lu [14]. ^177^Lu emits both β- and γ-radiation and is widely used as a theranostic pair with the positron-emitter ^68^Ga. The preferred radiometal chelator for potential theranostic applications is 1,4,7,10-tetraazacyclododecane-1,4,7,10-tetraacetic acid (DOTA), a macrocyclic bifunctional chelating agent which forms stable complexes with a variety of radiometals including ^68^Ga and ^177^Lu.

**Figure 1:**
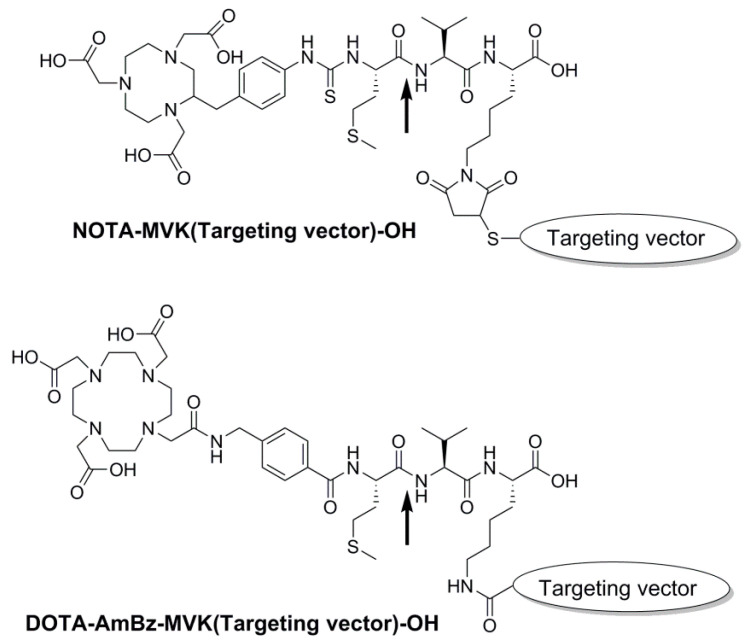
DOTA-AmBz-MVK(Targeting vector)-OH inspired by the reported design of NOTA-MVK(Targeting vector)-OH. The amide bond between Met-Val (pointed by an arrow) is recognized and cleaved by NEP.

In this study, we evaluated the MVK sequence as a cleavable tripeptide linker to reduce renal uptake of radiolabeled DOTA-conjugated peptides and peptidomimetics. We modified the original design, NOTA-MVK(Targeting vector)-OH, reported by Uehara et al. [12], and replaced the NOTA chelator with DOTA for potential radiolabeling with both imaging and radiotherapeutic isotopes (Figure 1). We also inserted an aminomethylbenzoyl (AmBz) group between DOTA and the MVK sequence to mimic the aminobenzyl group in the original design. The maleimide-thiol linkage at the Lys side chain was replaced with an amide linkage to enable the facile synthesis of the DOTA-conjugated peptides on solid phase.

**Figure 2:**
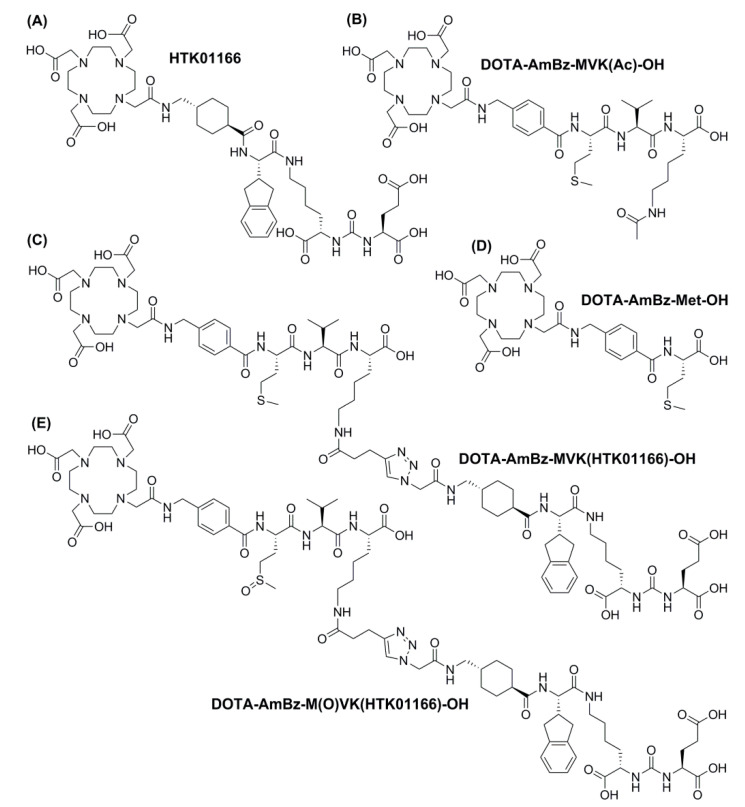
Chemical structures of (**A**) HTK01166, (**B**) DOTA-AmBz-MVK(Ac)-OH, (**C**) DOTA-AmBz-MVK(HTK01166)-OH, (**D**) DOTA-AmBz-Met-OH, and (**E**) DOTA-AmBz-M(O)VK(HTK01166)-OH.

We first synthesized the model compound, [^68^Ga]Ga-DOTA-AmBz-MVK(Ac)-OH (Figure 2), and confirmed that it could be recognized by the renal brush border enzymes and cleaved at the Met-Val amide bond. We conducted PET imaging and in vivo stability studies in mice and confirmed that the expected radio-metabolite [^68^Ga]Ga-DOTA-AmBz-Met-OH was rapidly excreted through the urinary pathway with minimal uptake in kidneys. We then replaced the acetyl group of [^68^Ga]Ga-DOTA-AmBz-MVK(Ac)-OH with the pharmacophore of HTK01166 (Figure 2), a peptidomimetic targeting the prostate-specific membrane antigen (PSMA), which is highly expressed in prostate cancer [15]. The targeting pharmacophore of [^68^Ga]Ga-HTK01166 was selected as it was shown previously to have very high renal uptake (147 %ID/g, 1 h p.i.) in mice. Since Met is prone to oxidation to generate methionine sulfoxide (Met(O) or M(O)) [16] and the resulting M(O)VK sequence might not be recognized and cleaved by the renal brush border enzyme, we also synthesized [^68^Ga]Ga-DOTA-AmBz-M(O)VK(HTK01166)-OH for comparison. Here we report the syntheses of [^68^Ga]Ga-DOTA-AmBz-MVK(Ac)-OH, [^68^Ga]Ga-DOTA-AmBz-MVK(HTK01166)-OH and [^68^Ga]Ga-DOTA-AmBz-M(O)VK(HTK01166)-OH, and the results of enzyme assay, PET imaging, ex vivo biodistribution, and in vivo stability studies to evaluate the applicability of using the MVK linker to reduce the renal uptake of radiolabeled DOTA-conjugated peptides and peptidomimetics.

## 2. Results

### 2.1. Chemistry and Radiochemistry

**Scheme 1:**
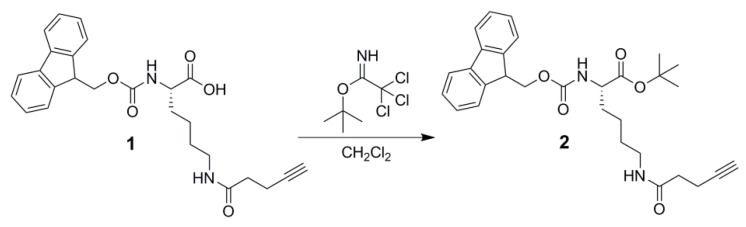
Synthesis of Fmoc-l-Lys(pentynoyl)-O*t*Bu (**2**).

Synthesis of Fmoc-Lys(pentynoyl)-O*t*Bu (**2**) is shown in Scheme 1. Fmoc-Lys(pentynoyl)-OH (**1**) prepared from literature procedures [17] was reacted with 2,2,2-trichloroacetimidate (2.2 equiv) in CH_2_Cl_2_. After overnight incubation at room temperature and purification by flash column chromatography, the desired product **2** was obtained in 79% yield.

**Scheme 2:**
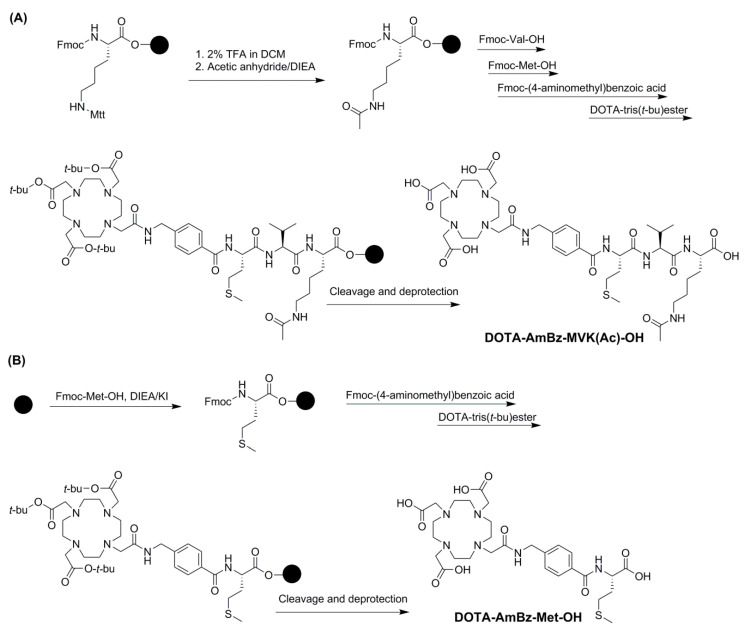
Synthesis of (**A**) DOTA-AmBz-MVK(Ac)-OH and (**B**) DOTA-AmBz-Met-OH. ● = resin.

**Scheme 3:**
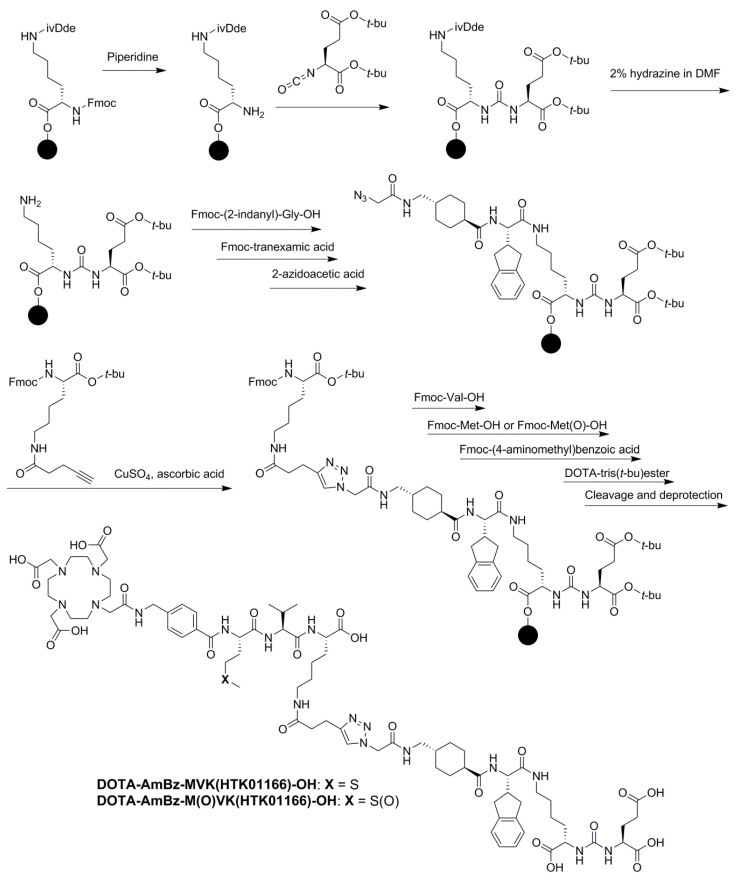
Synthesis of DOTA-AmBz-MVK(HTK01166)-OH and DOTA-AmBz-M(O)VK(HTK01166)-OH. ● = resin.

The DOTA-conjugated peptides and peptidomimetics including DOTA-AmBz-MVK(Ac)-OH, DOTA-AmBz-MVK(HTK01166)-OH, DOTA-AmBz-Met-OH, and DOTA-AmBz-M(O)VK(HTK01166)-OH (Figure 2) were assembled on solid phase (Scheme 2 and Scheme 3). After cleavage/deprotection with TFA and HPLC purification, these DOTA-conjugated peptides and peptidomimetics were obtained in 1–35% isolated yields. Their nonradioactive Ga-complexed standards were obtained by incubating the DOTA-conjugated peptides and peptidomimetics with excess GaCl_3_ in acetate buffer (0.1 M, pH 4.2) at 80 °C. After HPLC purification, the nonradioactive Ga-complexed standards were obtained in 16–78% isolated yields. Detailed HPLC conditions and retention times for the purification of the DOTA-conjugated peptides and peptidomimetics and their nonradioactive Ga-complexed standards are provided in Supplemental Tables S1 and S2 (see the Supplemental Materials). The identities of the DOTA-conjugated peptides and peptidomimetics and their nonradioactive Ga-complexed standards were confirmed by MS analysis.

^68^Ga labeling of the DOTA-conjugated peptides and peptidomimetics was conducted in HEPES buffer (2 M, pH 5.0) with microwave heating for 1 min. After HPLC purification, [^68^Ga]Ga-DOTA-AmBz-MVK(Ac)-OH, [^68^Ga]Ga-DOTA-AmBz-MVK(HTK01166)-OH and [^68^Ga]Ga-DOTA-AmBz-M(O)VK(HTK01166)-OH were obtained in 40–78% decay-corrected radiochemical yields with >2 GBq/µmol molar activity and >93% radiochemical purity. Detailed HPLC conditions and retention times for the purification and quality control of the ^68^Ga-labeled DOTA-conjugated peptides and peptidomimetics are provided in Supplemental Table S3 (see the Supplemental Materials).

### 2.2. In Vitro Enzyme Assays

**Figure 3:**
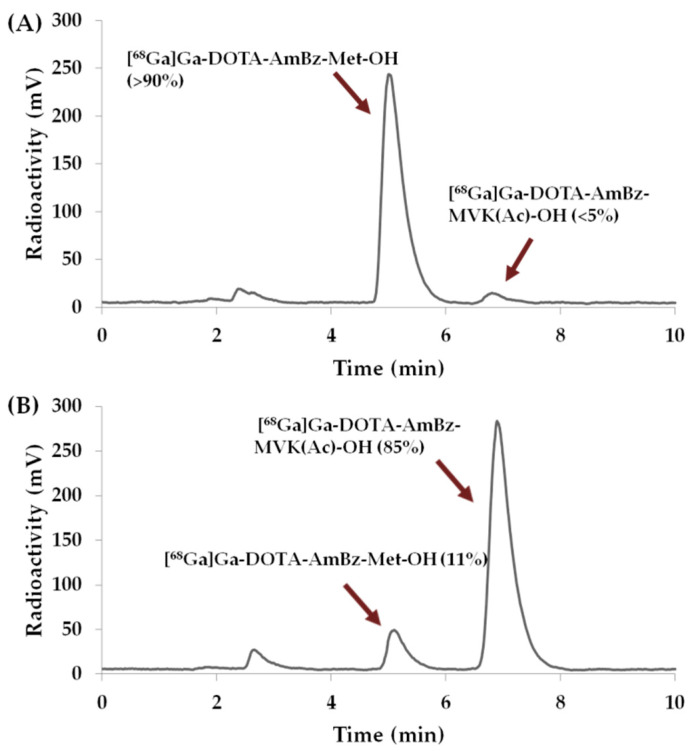
Radio-HPLC chromatograms of in vitro enzyme assay samples of [^68^Ga]Ga-DOTA-AmBz-MVK(Ac)-OH (**A**) without and (**B**) with the presence of phosphoramidon. HPLC conditions were 82/18 A/B at a flow rate of 2 mL/min; A: H_2_O containing 0.1% TFA; B: CH_3_CN containing 0.1% TFA; HPLC column: Luna C18, 5 µm particle size, 100 Å pore size, 250 × 4.6 mm.

In vitro enzyme assays revealed very efficient cleavage (>95%) of the ^68^Ga-labeled DOTA-conjugated linker, [^68^Ga]Ga-DOTA-AmBz-MVK(Ac)-OH, with the expected fragment [^68^Ga]Ga-DOTA-AmBz-Met-OH as the dominant radio-metabolite (>90%) (Figure 3A). The presence of the NEP inhibitor phosphoramidon significantly inhibited cleavage of [^68^Ga]Ga-DOTA-AmBz-MVK(Ac)-OH, with 85% of the recovered radioactivity as the intact tracer (Figure 3B).

**Figure 4:**
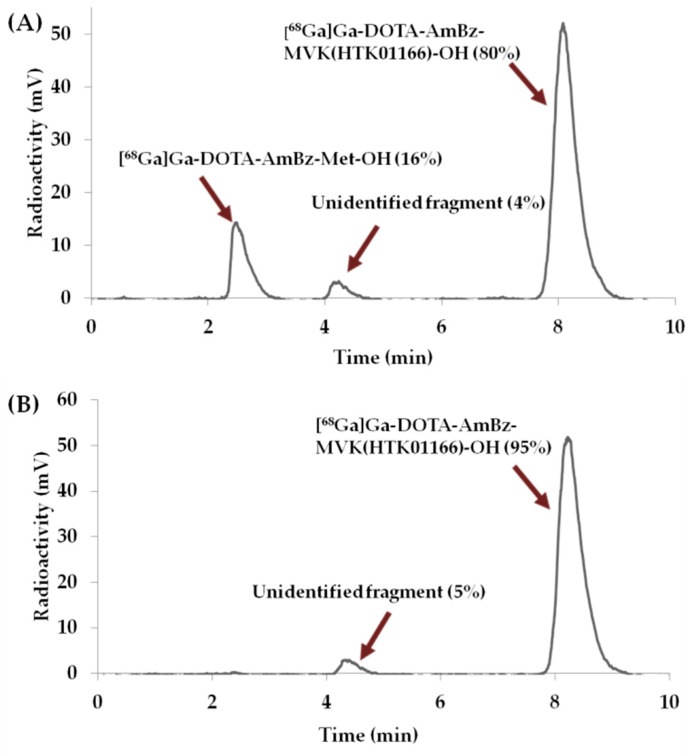
Radio-HPLC chromatograms of in vitro enzyme assay samples of [^68^Ga]Ga-DOTA-AmBz-MVK(HTK01166)-OH (**A**) without and (**B**) with the presence of phosphoramidon. HPLC conditions were 72/28 A/B at a flow rate of 2 mL/min; A: H_2_O containing 0.1% TFA; B: CH_3_CN containing 0.1% TFA; HPLC column: Luna C18, 5 µm particle size, 100 Å pore size, 250 × 4.6 mm.

[^68^Ga]Ga-DOTA-AmBz-MVK(HTK01166)-OH, a derivative of [^68^Ga]Ga-DOTA-AmBz-MVK(Ac)-OH by replacing the acetyl group with the PSMA-targeting motif Lys-urea-Glu and the lipophilic linker of HTK01166, was cleaved less effectively under the same assay conditions. After 1-h incubation, ~80% of [^68^Ga]Ga-DOTA-AmBz-MVK(HTK01166)-OH remained intact and ~16% of the recovered radioactivity was present as the expected radio-metabolite [^68^Ga]Ga-DOTA-AmBz-Met-OH (Figure 4A). An unidentified radio-metabolite with retention time at ~4.3 min accounted for ~4% of the recovered radioactivity. In the presence of phosphoramidon, the intact [^68^Ga]Ga-DOTA-AmBz-MVK(HTK01166)-OH fraction increased to ~95% and the formation of [^68^Ga]Ga-DOTA-AmBz-Met-OH was completely inhibited (Figure 4B).

**Figure 5:**
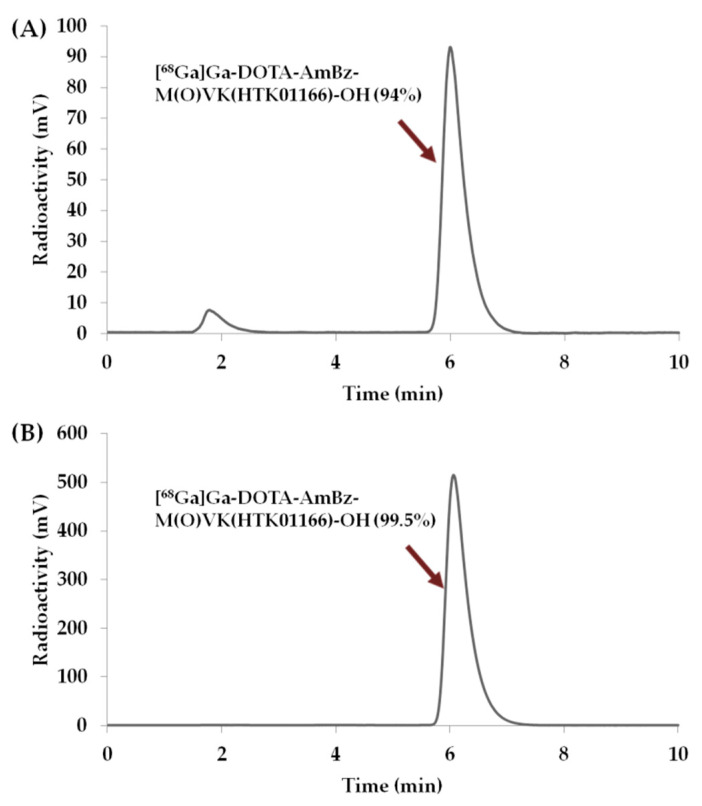
Radio-HPLC chromatograms of in vitro enzyme assay samples of [^68^Ga]Ga-DOTA-AmBz-M(O)VK(HTK01166)-OH (**A**) without and (**B**) with the presence of phosphoramidon. HPLC conditions were 76/24 A/B at a flow rate of 2 mL/min; A: H_2_O containing 0.1% TFA; B: CH_3_CN containing 0.1% TFA; HPLC column: Luna C18, 5 µm particle size, 100 Å pore size, 250 × 4.6 mm.

[^68^Ga]Ga-DOTA-AmBz-M(O)VK(HTK01166)-OH, the oxidized version of [^68^Ga]Ga-DOTA-AmBz-MVK(HTK01166)-OH was fairly stable under the same assay conditions with 94% and 99.5% remaining intact without and with the presence of phosphoramidon, respectively (Figure 5).

### 2.3. PET/CT Imaging and Ex Vivo Biodistribution Studies

**Figure 6:**
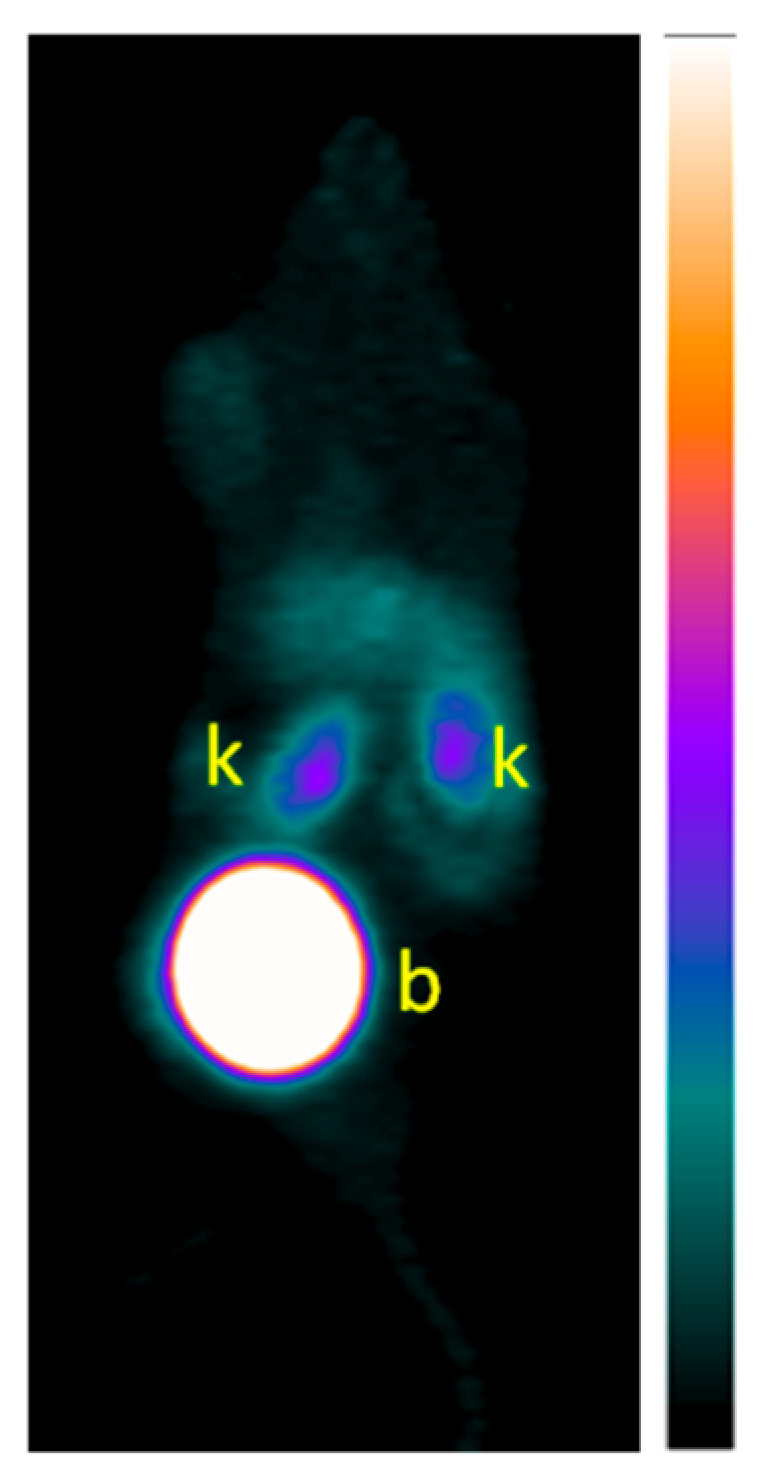
A representative maximum-intensity-projection PET image of [^68^Ga]Ga-DOTA-AmBz-MVK(Ac)-OH showing its rapid excretion predominantly via the renal pathway with minimal kidney retention (<2.5 %ID/g). The range of color bar is 0–5 %ID/g. k: kidney; b: bladder.

The pharmacokinetics of [^68^Ga]Ga-DOTA-AmBz-MVK(Ac)-OH was first evaluated in mice via PET/CT imaging studies. As shown in Figure 6, radioactivity from the injected [^68^Ga]Ga-DOTA-AmBz-MVK(Ac)-OH was excreted rapidly from blood pool and all background organs/tissues predominately via the renal pathway. At 1 h p.i., only kidneys and urinary bladder were clearly visualized in PET images, with low radioactivity (<2.5 %ID/g) retained in kidneys.

**Figure 7:**
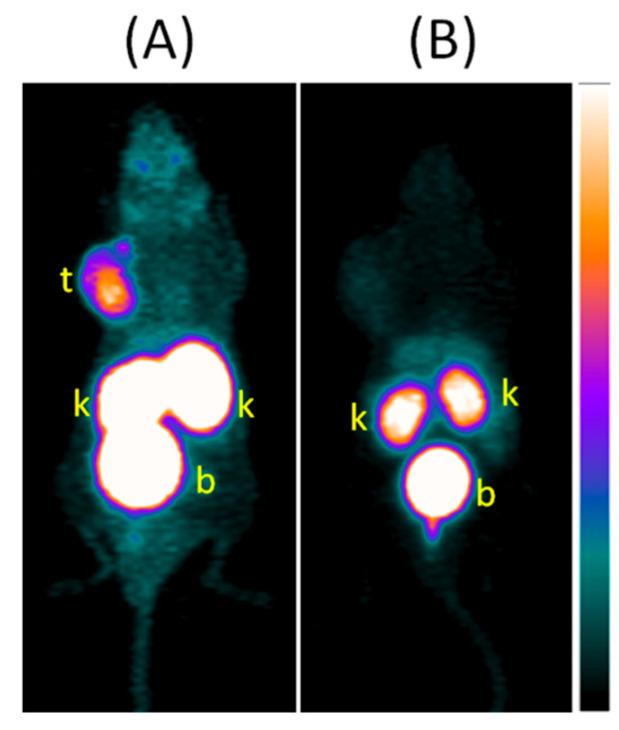
Representative maximum-intensity-projection PET images of [^68^Ga]Ga-DOTA-AmBz-MVK(HTK01166)-OH acquired at 1 h p.i. from LNCaP tumor-bearing mice (**A**) without and (**B**) with the co-injection of 2-PMPA (0.2 mg). The range of color bar is 0–5 %ID/g. t: tumor; k: kidney; b: bladder.

Next, we conducted the PET/CT imaging study of [^68^Ga]Ga-DOTA-AmBz-MVK(HTK01166)-OH in mice bearing PSMA-expressing LNCaP tumor xenografts to evaluate its pharmacokinetics and PSMA-targeting capability. As shown in Figure 7A, [^68^Ga]Ga-DOTA-AmBz-MVK(HTK01166)-OH was excreted quickly from background organs/tissues predominately via the renal pathway. At 1 h p.i., only urinary bladder and the PSMA-expressing kidneys and LNCaP tumor were clearly visualized in the PET images (Figure 7A). Co-injection of the PSMA inhibitor, 2-PMPA (2-(phosphonomethyl)-pentanedioic acid), blocked most of the uptake into the tumor and kidneys (Figure 7B).

**Figure 8:**
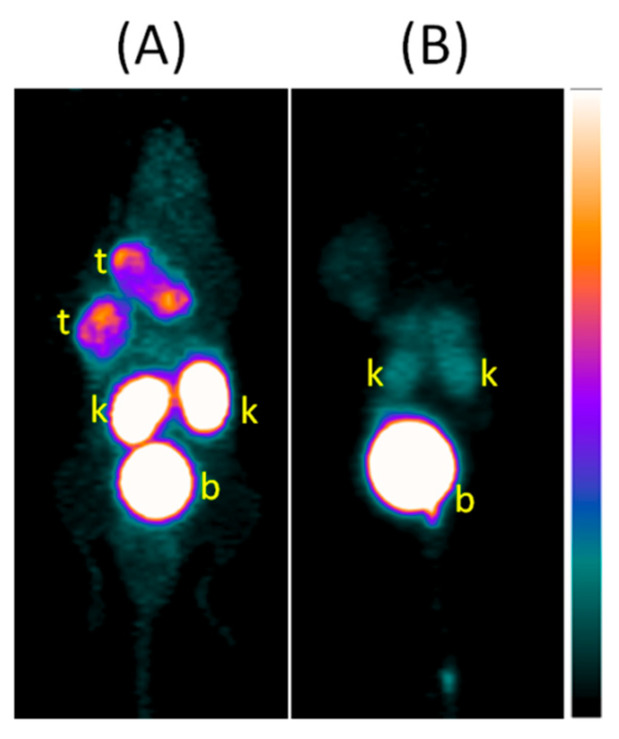
Representative maximum-intensity-projection PET/CT images of [^68^Ga]Ga-DOTA-AmBz-M(O)VK(HTK01166)-OH acquired at 1 h p.i. from LNCaP tumor-bearing mice (**A**) without and (**B**) with the co-injection of 2-PMPA (0.2 mg). The range of color bar is 0–5 %ID/g. t: tumor; k: kidney; b: bladder.

For comparison, the PET/CT imaging study was also conducted using [^68^Ga]Ga-DOTA-AmBz-M(O)VK(HTK01166)-OH, the sulfoxide analog of [^68^Ga]Ga-DOTA-AmBz-MVK(HTK01166)-OH. Similar to [^68^Ga]Ga-DOTA-AmBz-MVK(HTK01166)-OH, the excretion of [^68^Ga]Ga-DOTA-AmBz-M(O)VK(HTK01166)-OH was fast and predominately via the renal pathway (Figure 8A). At 1 h p.i., only urinary bladder, LNCaP tumor xenograft and kidneys were clearly visualized in PET images. Co-injection of 2-PMPA blocked most of the uptake in tumors and kidneys (Figure 8B), demonstrating the uptake was PSMA-mediated.

The ex vivo biodistribution studies were conducted for [^68^Ga]Ga-DOTA-AmBz-MVK(HTK01166)-OH and [^68^Ga]Ga-DOTA-AmBz-M(O)VK(HTK01166)-OH in LNCaP tumor-bearing mice, and the results are shown in Table 1. The ex vivo biodistribution data acquired at 1 h p.i. were consistent with the observations from their PET images (Figure 7A and Figure 8A, and Table 1) with low background in blood pool and non-target tissues/organs, good uptake in LNCaP tumor xenografts, and high uptake in kidneys. [^68^Ga]Ga-DOTA-AmBz-MVK(HTK01166)-OH and [^68^Ga]Ga-DOTA-AmBz-M(O)VK(HTK01166)-OH had similar uptake values (%ID/g) for the collected tissues/organs (blood: 0.55 ± 0.09 vs. 0.56 ± 0.13; pancreas: 0.60 ± 0.22 vs. 0.56 ± 0.24; spleen: 8.87 ± 2.63 vs. 11.5 ± 2.70; kidneys: 104 ± 12.2 vs. 89.7 ± 19.9; heart: 0.25 ± 0.12 vs. 0.23 ± 0.05; lung: 0.96 ± 0.12 vs. 0.88 ± 0.10; muscle: 0.21 ± 0.05 vs. 0.22 ± 0.05) and comparable tumor-to-background (blood, muscle and kidney) contrast ratios. Compared with the previously reported [^68^Ga]Ga-HTK01166 [15], [^68^Ga]Ga-DOTA-AmBz-M(O)VK(HTK01166)-OH showed 39% reduction in average kidney uptake (147 vs. 89.7 %ID/g, *p* = 0.025). [^68^Ga]Ga-DOTA-AmBz-MVK(HTK01166)-OH also showed 30% reduction in average kidney uptake (147 vs. 104 %ID/g), but the difference is not statistically significant (*p* = 0.055).

### 2.4. Quantification of Radio-Metabolites in Blood and Urine

Radio-metabolites of [^68^Ga]Ga-DOTA-AmBz-MVK(Ac)-OH and [^68^Ga]Ga-DOTA-AmBz-MVK(HTK01166)-OH in mouse blood and urine samples were analyzed by HPLC. The blood samples were collected at 5 min p.i. due to the fast excretion nature of the small radio-metabolites, whereas the urine samples were collected at 15 min p.i. to allow sufficient time for the accumulation of enough radio-metabolites for analysis.

**Figure 9:**
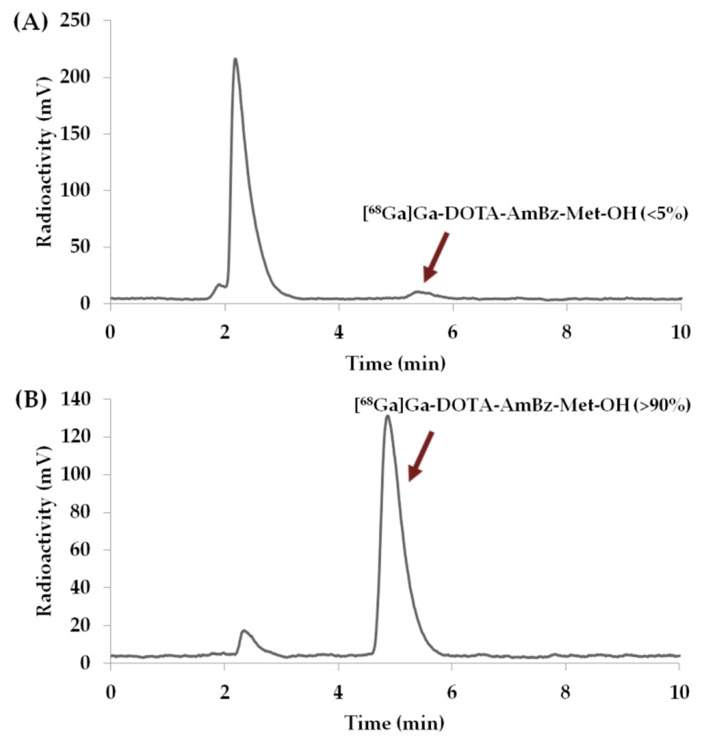
Representative radio-HPLC chromatograms of [^68^Ga]Ga-DOTA-AmBz-MVK(Ac)-OH obtained from mouse (**A**) blood and (**B**) urine samples collected at 5 and 15 min p.i., respectively. HPLC conditions were 82/18 A/B at a flow rate of 2 mL/min; A: H_2_O containing 0.1% TFA; B: CH_3_CN containing 0.1% TFA; HPLC column: Luna C18, 5 µm particle size, 100 Å pore size, 250 × 4.6 mm.

As shown in Figure 9A, there were mainly unidentified polar metabolites (retention time 1.5–3.0 min) present in the blood samples, but no intact [^68^Ga]Ga-DOTA-AmBz-MVK(Ac)-OH and only minimal amounts of the expected metabolite [^68^Ga]Ga-DOTA-AmBz-Met-OH (<5%) was detected. On the contrary, >90% of the radioactivity presented in the urine samples was [^68^Ga]Ga-DOTA-AmBz-Met-OH (Figure 9B).

**Figure 10:**
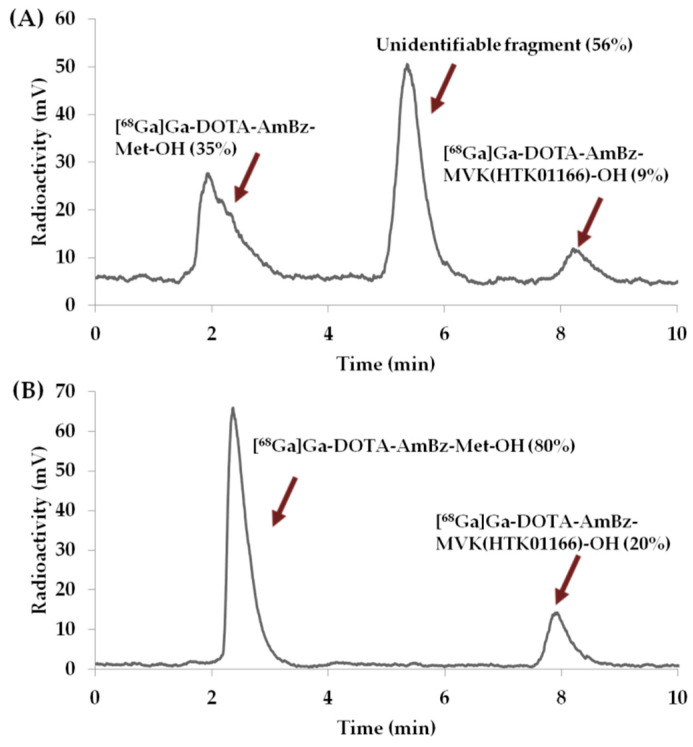
Representative radio-HPLC chromatograms of [^68^Ga]Ga-DOTA-AmBz-MVK(HTK01166)-OH obtained from mouse (**A**) blood and (**B**) urine samples collected at 5 and 15 min p.i., respectively. HPLC conditions were 72/28 A/B at a flow rate of 2 mL/min; A: H_2_O containing 0.1% TFA; B: CH_3_CN containing 0.1% TFA; HPLC column: Luna C18, 5 µm particle size, 100 Å pore size, 250 × 4.6 mm.

Analysis of blood samples from mice injected with [^68^Ga]Ga-DOTA-AmBz-MVK(HTK01166)-OH showed only 9% remaining intact and 35% of the tracer was metabolized to the expected fragment [^68^Ga]Ga-DOTA-AmBz-Met-OH (Figure 10A). However, a major unidentified radio-metabolite, accounting for 56% of the recovered radioactivity, was also observed. Analysis of the urine samples revealed that only 20% of the recovered radioactivity was presented as the intact tracer and the remaining 80% was the expected fragment [^68^Ga]Ga-DOTA-AmBz-Met-OH (Figure 10B).

## 3. Discussion

The potential of NEP to recognize and cleave specific sequences has been exploited by various groups in the recent years to reduce kidney uptake of radiopharmaceuticals [1,13,18]. This is attributable to the abundant expression of NEP in the brush border membrane lining primarily of the proximal convoluted tubules of the juxtamedullary nephrons [11]. The mechanism underlying degradation of certain specific linker sequences has been well elucidated [1]. The type of amino acid in the radio-metabolite(s) as well as the radiometal chelate used plays a critical role in deciding the kidney residence time of the generated radio-metabolite(s).

NEP is a type-II integral membrane glycoprotein with metalloendopeptidase activity, and also presents an even better carboxydipeptidase activity when the two situations are possible [19]. To apply this strategy to reduce the kidney uptake of radiolabeled peptides and peptidomimetics, we modified the original design as shown in Figure 2. We followed the same design with the targeting vector conjugated to the cleavable linker at the Lys side chain. Such design preserves the free carboxylic group of Lys and enhances the cleavage of the MVK linker by NEP via its carboxydipeptidase activity.

We first synthesized the model tracer [^68^Ga]Ga-DOTA-AmBz-MVK(Ac)-OH with an acetyl group coupled to the ε-amino group of Lys to provide the amide linkage which would be present when a targeting vector is coupled to this linker. Enzyme assays using the brush border membrane vesicles (BBMVs) extracted from mouse kidneys confirmed that [^68^Ga]Ga-DOTA-AmBz-MVK(Ac)-OH can be efficiently cleaved (>95%) by renal brush border enzymes (Figure 3), and the expected [^68^Ga]Ga-DOTA-AmBz-Met-OH was identified as the major radio-metabolite (>90%). Co-incubation with the NEP inhibitor phosphoramidon greatly enhanced the stability of [^68^Ga]Ga-DOTA-AmBz-MVK(Ac)-OH against renal brush border enzymes (~85% remaining intact), indicating the cleavage of [^68^Ga]Ga-DOTA-AmBz-MVK(Ac)-OH into [^68^Ga]Ga-DOTA-AmBz-Met-OH was mediated by NEP. These data confirmed the success of our modifications on the original design (NOTA→DOTA, aminobenzyl→aminomethylbenzoyl, and maleimide-thiol linkage→amide linkage, Figure 1), and the resulting [^68^Ga]Ga-DOTA-AmBz-MVK(Ac)-OH was still recognized and cleaved by NEP at the same Met-Val amide bond.

We then conducted PET imaging and in vivo stability studies in mice to confirm that [^68^Ga]Ga-DOTA-AmBz-MVK(Ac)-OH can be metabolized in vivo into [^68^Ga]Ga-DOTA-Met-OH, and low retention of [^68^Ga]Ga-DOTA-AmBz-Met-OH in kidneys. This is vital for the success of this modified strategy as it depends on the generation of the expected radio-metabolite [^68^Ga]Ga-DOTA-AmBz-Met-OH and most importantly [^68^Ga]Ga-DOTA-AmBz-Met-OH needs to have low kidney retention too. As shown in Figure 6, [^68^Ga]Ga-DOTA-AmBz-MVK(Ac)-OH was excreted rapidly and predominately via the renal pathway with low kidney retention (<2.5 %ID/g at 1 h p.i.). The blood samples of [^68^Ga]Ga-DOTA-AmBz-MVK(Ac)-OH revealed no presence of the intact tracer and a small fraction of the expected radio-metabolite [^68^Ga]Ga-DOTA-AmBz-Met-OH (<5%, Figure 9A). This indicates that [^68^Ga]Ga-DOTA-AmBz-MVK(Ac)-OH was cleared rapidly from the blood pool either as its intact form or as radio-metabolite(s). Besides kidneys, NEP is also present in some tissues although in a much lower expression level. Therefore, the cleavage of [^68^Ga]Ga-DOTA-AmBz-MVK(Ac)-OH by NEP present in other tissues cannot be ruled out. Analysis of the urine samples revealed no presence of [^68^Ga]Ga-DOTA-AmBz-MVK(Ac)-OH and [^68^Ga]Ga-DOTA-AmBz-Met-OH was presented as the major radio-metabolite (>90%). These data suggest that [^68^Ga]Ga-DOTA-AmBz-Met-OH had low retention in kidneys and any intact [^68^Ga]Ga-DOTA-AmBz-MVK(Ac)-OH excreted through kidneys was metabolized presumably by renal brush border enzymes mainly into [^68^Ga]Ga-DOTA-AmBz-Met-OH.

After confirming the in vivo cleavage of [^68^Ga]Ga-DOTA-AmBz-MVK(Ac)-OH into [^68^Ga]Ga-DOTA-AmBz-Met-OH, and the low kidney retention of the [^68^Ga]Ga-DOTA-AmBz-Met-OH, we next tested this modified design with a PSMA-targeting vector coupled to the Lys side chain. PSMA has become a very promising imaging and therapeutic target for the management of prostate cancer. However, due to a high expression level of PSMA in kidneys, high and sustained renal uptake of PSMA-targeting radioligands are constantly observed, leading to suboptimal detection sensitivity for lesions adjacent to kidneys, and concerns for renal toxicity when radiotherapeutic agents are used. The idea of incorporating the MVK cleavable linker to a radiolabeled DOTA-conjugated PSMA-targeting radioligand is to have the radioligand cleaved by the renal brush border enzymes when it is excreted through kidneys. Since the expected radiometal-complexed DOTA-AmBz-Met-OH is not retained in kidneys, the overall renal uptake of the PSMA-targeting radioligand containing the MVK cleavable linker will be reduced.

Enzyme assays revealed that unlike the instability of [^68^Ga]Ga-DOTA-AmBz-MVK(Ac)-OH against renal brush border enzymes, [^68^Ga]Ga-DOTA-AmBz-MVK(HTK01116)-OH was relatively stable with 80% remaining intact and 16% converted to the expected radio-metabolite [^68^Ga]Ga-DOTA-AmBz-Met-OH under the same assay conditions (Figure 4). The enhanced stability could be due to the steric hindrance introduced by replacing the acetyl group with the much bulkier HTK01166 motif. Further enhancement in stability achieved by co-incubation with the NEP inhibitor phosphoramidon indicates that [^68^Ga]Ga-DOTA-AmBz-MVK(HTK01116)-OH was cleaved mainly by NEP into [^68^Ga]Ga-DOTA-AmBz-Met-OH.

PET imaging studies showed that [^68^Ga]Ga-DOTA-AmBz-MVK(HTK01166)-OH and its oxidized version [^68^Ga]Ga-DOTA-AmBz-M(O)VK(HTK01166)-OH had very similar distribution patterns: low background, high kidney retention and good tumor visualization (Figure 7 and Figure 8). The observed PET images were consistent with the ex vivo biodistribution data showing comparable uptake for all collected organs/tissues (Table 1). The brain uptake (≤0.03 %ID/g) of both tracers was negligible indicating that they cannot freely cross the blood-brain barrier. Their fast blood clearance (~0.55 %ID/g at 1 h p.i.) suggests that the [^68^Ga]Ga-DOTA complex was stable as free ^68^Ga would be captured by transferrins leading to prolonged retention in blood pool [20]. Minimal uptake (≤0.60 %ID/g) of both tracers in liver and intestines indicates that they were excreted mainly by the renal pathway. Higher uptake was observed in PSMA-expressing LNCaP tumors (~4 %ID/g), kidneys (90–104 %ID/g) and spleen (8.9–11.5 %ID/g) suggesting that the uptake of both tracers in these tissues was PSMA-mediated [15]. This was further confirmed by PET imaging studies as co-injection of the PSMA inhibitor, 2-PMPA (0.2 mg), reduced the tumor uptake of both tracers to the background level (Figure 7 and Figure 8).

The average kidney uptake of [^68^Ga]Ga-DOTA-AmBz-MVK(HTK01166)-OH was lower than that of the previously reported [^68^Ga]Ga-HTK01166 (104 vs. 147 %ID/g), suggesting the insertion of the cleavable MVK linker might be a useful strategy to reduce kidney uptake of radiopharmaceuticals. However, although statistically not significant (*p* = 0.15), a slightly higher kidney retention was observed for mice injected with [^68^Ga]Ga-DOTA-AmBz-MVK(HTK01166)-OH (104 ± 12.2 %ID/g) than for mice injected with [^68^Ga]Ga-DOTA-AmBz-MV(O)K(HTK01166)-OH (89.7 ± 19.3 %ID/g). Since [^68^Ga]Ga-DOTA-AmBz-MVK(HTK01166)-OH contains a cleavable MVK linker, whereas [^68^Ga]Ga-DOTA-AmBz-M(O)VK(HTK01166)-OH contains a non-cleavable M(O)VK linker, we would expect a much lower kidney uptake from [^68^Ga]Ga-DOTA-AmBz-MVK(HTK01166)-OH. These discrepant data suggest that there might be some unknown mechanism(s) causing the higher kidney retention of [^68^Ga]Ga-DOTA-AmBz-MVK(HTK01166)-OH. In addition, we noticed that while the kidney retention (1.3 %ID/g) in the mouse injected with [^68^Ga]Ga-DOTA-AmBz-M(O)VK(HTK01166)-OH and 2-PMPA was minimal (Figure 8B), there was significantly higher kidney uptake (4.2 %ID/g) in the mouse injected with [^68^Ga]Ga-DOTA-AmBz-MVK(HTK01166)-OH and 2-PMPA (Figure 7B).

The retained kidney uptake was unlikely to have resulted from the intact [^68^Ga]Ga-DOTA-AmBz-MVK(HTK01166)-OH, as the mouse was co-injected with excess 2-PMPA to block PSMA. The retained uptake was unlikely to have resulted from the expected radio-metabolite [^68^Ga]Ga-DOTA-AmBz-Met-OH either, as we have shown that no significant kidney retention was observed in the mouse injected with [^68^Ga]Ga-DOTA-AmBz-MVK(Ac)-OH (Figure 6). The only possibility for the kidney retention would be unidentified radio-metabolite(s), which cannot bind PSMA but can be retained in kidneys. Therefore, the in vivo stability study was further conducted for [^68^Ga]Ga-DOTA-AmBz-MVK(HTK01166)-OH to discern the cause of its higher kidney retention in mice.

As shown in Figure 10A, unlike the good stability observed in enzyme assays, [^68^Ga]Ga-DOTA-AmBz-MVK(HTK01166)-OH was rapidly metabolized in vivo with only 9% of the tracer remaining intact in the blood at 5 min p.i. However, the expected radio-metabolite [^68^Ga]Ga-DOTA-AmBz-Met-OH was accounted for only 35% of the recovered radioactivity, while 56% consists of an unidentified radio-metabolite. Interestingly, in urine samples, only [^68^Ga]Ga-DOTA-AmBz-MVK(HTK01166)-OH (20%) and [^68^Ga]Ga-DOTA-AmBz-Met-OH (80%) were detected, and there was no presence of the unidentified radio-metabolite (Figure 10B). The absence of the unidentified radio-metabolite in urine samples suggests that it might be retained in kidneys. This would explain the higher kidney uptake of [^68^Ga]Ga-DOTA-AmBz-MVK(HTK01166)-OH than [^68^Ga]Ga-DOTA-AmBz-M(O)VK(HTK01166)-OH (Table 1), as well as its higher kidney retention when co-injected with 2-PMPA (Figure 7 and Figure 8). This would also explain the insufficient reduction (~30%) in kidney uptake when compared [^68^Ga]Ga-DOTA-AmBz-MVK(HTK01166)-OH and [^68^Ga]Ga-HTK01166 [15], which is far less than the ~80% and ~67% reduction in kidney uptake reported by Uehara et al. [12] and Zhang et al. [13], respectively, using the radiolabeled NOTA-MVK(Targeting vector)-OH design. The insufficient reduction reported here using the DOTA-AmBz-MVK(Targeting vector)-OH design is likely caused by the unidentified radio-metabolite that could be trapped in kidneys. Therefore, further optimization of the design of DOTA-conjugated cleavable linkers should avoid the generation of radio-metabolites that could be trapped in kidneys and cause high and sustained kidney uptake.

The identity of the unidentified fragment remains unknown (Figure 10). This is because the core structure of HTK01166 contains no amide bonds formed by two natural amino acids. Moreover, the data from our [^68^Ga]Ga-DOTA-AmBz-MVK(Ac)-OH, [^68^Ga]Ga-DOTA-AmBz-M(O)VK(HTK01166)-OH and the previously reported [^68^Ga]Ga-NOTA- MVK-conjugated antibody fragment [12] and Exendin 4 [13], did not suggest the cleavage of the Val-Lys amide bond. Therefore, we did not expect cleavage of [^68^Ga]Ga-DOTA-AmBz-MVK(HTK01166)-OH at locations other than the Met-Val amide bond, and more investigations are needed to verify the identity of this unknown radio-metabolite.

To conclude, we showed that replacing NOTA and the aminobenzyl group in the reported NOTA-MVK linker with DOTA and AmBz, respectively, generated the model compound [^68^Ga]Ga-DOTA-AmBz-MVK(Ac)-OH, which was still recognized and specifically cleaved at the Met-Val amide bond by NEP. Coupling a bulkier PSMA-targeting vector to the side chain of Lys in DOTA-AmBz-MVK enhanced its stability against NEP, but possibly also rendered its vulnerability against other enzyme(s) as evident by the formation of an unidentified radio-metabolite that could be retained in kidneys. Nevertheless, the renal uptake of the resulting [^68^Ga]Ga-DOTA-AmBz-MVK(HTK01166)-OH was still lower than that of [^68^Ga]Ga-HTK01166. These data demonstrated that MVK could be a promising cleavable linker for use to reduce renal uptake of radiolabeled DOTA-conjugated tumor-targeting peptides and peptidomimetics. This strategy can be used to enhance detection sensitivity of the imaging agents for lesions adjacent to kidneys, and improve the tumor-to-kidney absorbed dose ratio for the radiotherapeutic agents.

## 4. Materials and Methods

### 4.1. General Methods

Fmoc-l-Lys(pentynoyl)-OH (**1**) was synthesized according to the literature procedures [17]. Brush border membrane vesicles (BBMVs) were extracted from mouse kidneys following literature procedures [21]. All other chemicals were procured from commercial sources and used without further purification. All peptides and peptidomimetics were synthesized either on an AAPPTec (Louisville, KY, USA) Endeavor 90 peptide synthesizer or a CEM (Matthews, NC, USA) Liberty Blue™ automated microwave peptide synthesizer. Purification and quality control of radiolabeling precursor, nonradioactive Ga-complexed standards and ^68^Ga-labeled peptides and peptidomimetics were performed on Agilent (Santa Clara, CA, USA) HPLC systems equipped with a model 1200 quaternary pump, a model 1200 UV absorbance detector (set at 220 nm), and a Bioscan (Washington, DC, USA) NaI scintillation detector. The operation of Agilent HPLC systems was controlled using the Agilent ChemStation software. HPLC columns used were a semipreparative column (Luna C18, 5 µm particle size, 100 Å pore size, 250 × 10 mm) and an analytical column (Luna C18, 5 µm particle size, 100 Å pore size, 250 × 4.6 mm) from Phenomenex (Torrance, CA, USA). The HPLC solvents were A: H_2_O containing 0.1% TFA; B: CH_3_CN containing 0.1% TFA. The collected HPLC eluates containing the desired peptides were lyophilized using a Labconco (Kansas City, MO, USA) FreeZone 4.5 Plus freeze drier. ^1^H-NMR spectrum was acquired using an AVANCE Bruker 400 MHz NMR spectrometer equipped with BBI probe with Z gradients. Mass analyses were performed using an AB SCIEX (Framingham, MA, USA) 4000 QTRAP mass spectrometer system with an ESI ion source. C18 Sep-Pak cartridges (1 cm^3^, 50 mg) were obtained from Waters (Milford, MA, USA). ^68^Ga was eluted from an iThemba Laboratories (Somerset West, South Africa) generator and purified according to the previously published procedures using a DGA resin column from Eichrom Technologies LLC (Lisle, IL, USA) [22,23]. Radioactivity of ^68^Ga-labeled peptides and peptidomimetics was measured using a Capintec (Ramsey, NJ, USA) CRC-25R/W dose calibrator. PET/CT imaging was performed using a Siemens Inveon (Knoxville, TN, USA) micro PET/CT scanner. The radioactivity of mouse tissues collected from biodistribution studies was counted using a PerkinElmer (Waltham, MA, USA) Wizard2 2480 automatic gamma counter.

### 4.2. Synthesis of Fmoc-l-Lys(pentynoyl)-OtBu (2)

Fmoc-l-Lys(pentynoyl)-OH (**1**) (2.05 g, 4.6 mmol) in CH_2_Cl_2_ (20 mL) was added *t*-butyl 2,2,2-trichloroacetimidate (2.18 g, 10 mmol). The resulting mixture was stirred at RT for 22 h, and purified by flash column chromatography eluted with 1:1 ethyl acetate/hexane to 100% ethyl acetate. The desired product **2** was obtained as a white solid (1.82 g, 79%). ^1^H-NMR (400 MHz, CDCl_3_) δ ppm: 1.39–2.08 (m, 7H), 1.47 (s, 9H), 2.37 (t, *J* = 7.0 Hz, 2H), 2.52 (dt, *J* = 2.2, 7.0 Hz, 2H). 3.28 (m, 2H), 4.22 (t, *J* = 7.0 Hz, 2H), 4.39 (m, 2H), 5.42 (d, *J* = 8.0 Hz, 1H), 5.90 (bs, 1H), 7.31 (dt, *J* = 1.0, 7.4 Hz, 2H), 7.40 (t, *J* = 7.5 Hz, 2H), 7.60 (d, *J* = 7.4 Hz, 2H), 7.77 (d, *J* = 7.5 Hz, 2H). ESI-MS: calculated [M + H]^+^ for Fmoc-l-Lys(pentynoyl)-OtBu C_30_H_36_N_2_O_5_ 505.3; found 506.0.

### 4.3. Synthesis of DOTA-Conjugated Precursors

#### 4.3.1. Synthesis of DOTA-AmBz-MVK(Ac)-OH

For synthesizing the DOTA-conjugated linker DOTA-AmBz-MVK(Ac)-OH, Fmoc-Lys(Mtt) wang resin (0.05 mmol scale, 0.5–0.8 mmol/g loading) was first swollen using DMF. The Mtt protecting group was removed using 2% TFA in DCM for 30 min (5 min, 6 times) and neutralized using DIEA in DMF. The free amine was acetylated using acetic anhydride (20 equiv)/DIEA (20 equiv)/DMF. Fmoc on Lys was then deprotected using 20% piperidine in DMF and coupled with Fmoc-Val-OH (5 equiv) in the presence of activators HATU/HOAt (5 equiv) and DIEA (10 equiv) in DMF. Further elongation of the peptide chain was carried out by repeating the Fmoc deprotection and coupling steps. Fmoc-Met-OH, Fmoc-(4-aminomethyl)benzoic acid, and finally DOTA-tris(*t*-bu)ester (tri-*t*-butyl 1,4,7,10-tetraazacyclododecane-1,4,7,10-tetraacetate) were coupled in that order. The DOTA conjugated peptide was cleaved off the resin and simultaneously deprotected using TFA:H_2_O:triisopropylsilane (TIS):2,2′-(ethylenedioxy)diethanethiol (DODT) for 1.5 h at RT, in a 92.5:2.5:2.5:2.5 ratio. The cleaved peptide was filtered and precipitated with cold diethyl ether before purification using the semipreparative HPLC column. HPLC conditions were 84/16 A/B at a flow rate of 4.5 mL/min. The retention time of DOTA-AmBz-MVK(Ac)-OH was 14.1 min. Eluates containing the desired peptide were collected, pooled, and lyophilized. The isolated yield was 12%. ESI-MS: calculated [M + H]^+^ for DOTA-AmBz-MVK(Ac)-OH C_42_H_67_N_9_O_13_S 938.5; found 938.4.

#### 4.3.2. Synthesis of DOTA-AmBz-MVK(HTK01166)-OH

For synthesizing the PSMA-targeting DOTA-AmBz-MVK(HTK01166)-OH the PSMA-targeting motif (Lys-urea-Glu) and the lipophilic linker ((2-indanyl)-Gly-tranexamic acid) of HTK01166 were first assembled on solid phase following our previously published procedures [15]. Briefly, Fmoc-Lys(ivDde) wang resin (0.1 mmol scale, 0.58 mmol/g loading) was swollen using DMF. The isocyanate derivative of di-*tert*-butyl ester of glutamate (5 equiv) was prepared according to literature procedures [24]. The isocyante derivative was then added to Fmoc-deprotected Lys(ivDde) wang resin and the reaction mixture was allowed to shake overnight. Next, the ivDde protecting group was removed using 2% hydrazine in DMF (5 mL, five times, 5 min). Subsequent couplings of Fmoc-(2-indanyl)-Gly-OH, Fmoc-tranexamic acid and 2-azidoacetic acid were conducted using standard Fmoc chemistry. Next, Fmoc-Lys(pentynoyl)-O*t*Bu (**2**), (0.5 mmol) was clicked onto the azido group using CuSO_4_ (0.05 mmol) and ascorbic acid (0.25 mmol). After the click reaction, further peptide elongation was continued using Fmoc-Val-OH, Fmoc-Met-OH, Fmoc-(4-aminomethyl)benzoic acid, and finally, DOTA-tris(*t*-bu)ester. Peptide cleavage and deprotection was performed using TFA:H_2_O:TIS:DODT for 1.5 h at RT in a 92.5:2.5:2.5:2.5 ratio. The cleaved peptide was filtered and precipitated with cold diethyl ether before purification using the semipreparative HPLC column. HPLC conditions were 73/27 A/B at a flow rate of 4.5 mL/min. The retention time of DOTA-AmBz-MVK(HTK01166)-OH was 12.6 min. Eluates containing the desired peptide were collected, pooled, and lyophilized. The isolated yield was 1%. ESI-MS: calculated [M + H]^+^ for DOTA-AmBz-MVK(HTK01166)-OH C_78_H_115_N_17_O_23_S 1690.8; found 1690.9.

#### 4.3.3. Synthesis of DOTA-AmBz-M(O)VK(HTK01166)-OH

DOTA-AmBz-M(O)VK(HTK01166)-OH, the oxidized (sulfoxide) version of DOTA-AmBz-MVK(HTK01166)-OH was synthesized as a control using a similar synthetic and purification strategy as for DOTA-AmBz-MVK(HTK01166)-OH. For this purpose, Fmoc-Met-OH was replaced with Fmoc-Met(O)-OH. The HPLC conditions were 74/26 A/B at a flow rate of 4.5 mL/min. The retention time and isolated yield were 8.3 min and 2%, respectively. ESI-MS: calculated [M + H]^+^ for DOTA-AmBz-M(O)VK(HTK01166)-OH C_78_H_115_N_17_O_24_S 1706.8; found 1706.9.

#### 4.3.4. Synthesis of DOTA-AmBz-Met-OH

For synthesizing the expected cleaved fragment DOTA-AmBz-Met-OH, ClTCP(Cl)ProTide resin (0.1 mmol scale, 0.48 mmol/g loading) was swollen in DMF. Fmoc-Met-OH (5 equiv) in DMF containing 1 M DIEA/0.125M KI was coupled to the resin. After elongation with Fmoc-(4-aminomethyl)benzoic acid and DOTA-tris(*t*-bu)ester, the peptide was cleaved off the resin using TFA:H_2_O:TIS:DODT for 1.5 h at RT in a 92.5:2.5:2.5:2.5 ratio and purified using the semipreparative HPLC column. The HPLC conditions were 83/17 A/B at a flow rate of 4.5 mL/min. The retention time was 10.2 min, and the isolated yield was 35%. ESI-MS: calculated [M + H]^+^ for DOTA-AmBz-Met-OH C_29_H_44_N_6_O_10_S 669.3; found 669.0.

### 4.4. Synthesis of Nonradioactive Ga-Complexed Standards

Ga-DOTA-AmBz-MVK(Ac)-OH, Ga-DOTA-AmBz-Met-OH, Ga-DOTA-AmBz-MVK(HTK01166)-OH and Ga-DOTA-AmBz-M(O)VK(HTK01166)-OH were prepared by incubating the DOTA-conjugated peptides and peptidomimetics with GaCl_3_ (5 equiv) in NaOAc buffer (0.1 M, 300–500 μL, pH 4.2) at 80 °C for 15 min. The reaction mixtures were directly purified using the semipreparative HPLC column. The eluates containing the desired product were collected, pooled, and lyophilized.

For Ga-DOTA-AmBz-MVK(Ac)-OH the HPLC conditions were 82/18 A/B at a flow rate of 4.5 mL/min. The retention time and isolated yield were 11.2 min and 78%, respectively. ESI-MS: calculated [M + H]^+^ for Ga-DOTA-AmBz-MVK(Ac)-OH C_42_GaH_65_N_9_O_13_S 1005.4; found 1004.4.

For Ga-DOTA-AmBz-MVK(HTK01166)-OH the HPLC conditions were 74/26 A/B at a flow rate of 4.5 mL/min. The retention time and isolated yield were 8.0 min and 16%, respectively. ESI-MS: calculated [M + H]^+^ for Ga-DOTA-AmBz-MVK(HTK01166)-OH C_78_GaH_113_N_17_O_23_S 1757.7; found 1758.4.

For Ga-DOTA-AmBz-M(O)VK(HTK01166)-OH the HPLC conditions were 74/26 A/B at a flow rate of 4.5 mL/min. The retention time and isolated yield were 10.8 min and 35%, respectively. ESI-MS: calculated [M + 2H]^2+^ for Ga-DOTA-AmBz-M(O)VK(HTK01166)-OH C_78_GaH_113_N_17_O_24_S 887.4; found 887.5.

For Ga-DOTA-AmBz-Met-OH, the HPLC conditions were 83/17 at a flow rate of 4.5 mL/min. The retention time and the isolated yield were 10.5 min and 72%, respectively. ESI-MS: calculated [M + H]^+^ for Ga-DOTA-AmBz-Met-OH C_29_GaH_42_N_6_O_10_S 736.2; found 736.1.

### 4.5. Synthesis of ^68^Ga-Labeled Peptides and Peptidomimetics

Purification of ^68^Ga eluated from ^68^Ge/^68^Ga generator and labeling experiments were performed following our previously published procedures [22,23]. Purified ^68^Ga in 0.5 mL of water was added into a 4-mL glass vial preloaded with 0.7 mL of HEPES buffer (2 M, pH 5.0) and 25 μg of the precursor. The radiolabeling reaction was carried out under microwave heating for 1 min. The reaction mixtures were purified using the semipreparative HPLC column and eluted with 84/16 A/B for [^68^Ga]Ga-DOTA-AmBz-MVK(Ac)-OH, 74/26 A/B for [^68^Ga]Ga-DOTA-AmBz-MVK(HTK01166)-OH, and 23% CH_3_CN and 0.1% HCOOH in H_2_O for [^68^Ga]Ga-DOTA-AmBz-M(O)VK(HTK01166)-OH at a flow rate of 4.5 mL/min. The retention times for [^68^Ga]Ga-DOTA-AmBz-MVK(Ac)-OH, [^68^Ga]Ga-DOTA-AmBz-MVK(HTK01166)-OH and [^68^Ga]Ga-DOTA-AmBz-M(O)VK(HTK01166)-OH were 24.3, 26.1 and 15.7 min, respectively. The eluate fraction containing the radiolabeled product was collected, diluted with water (50 mL), and passed through a C18 Sep-Pak cartridge that was prewashed with ethanol (10 mL) and water (10 mL). After washing the C18 Sep-Pak cartridge with water (10 mL), the ^68^Ga-labeled product was eluted off the cartridge with ethanol (0.4 mL) and diluted with saline for all the in vitro enzyme assays, PET imaging, ex vivo biodistribution and in vivo stability studies.

### 4.6. In Vitro Enzyme Assay

The enzymatic recognition of synthesized peptides and peptidomimetics was determined by incubating the ^68^Ga-labeled peptides and peptidomimetics with the extracted BBMVs at 37 °C for 1 h. For the assay, aliquots (25 µL) of the enzyme solution (1.52 mg/mL) and enzyme buffer (250 mM NaCl, 57.5 mM Tris-base; adjusted to a pH 7.5) were mixed in a 96-well clear bottom plate and incubated at 37 °C for 10 min. The mixtures also contained 100 ppm ascorbic acid to prevent oxidation of the Met residue in the tested peptides and peptidomimetics during the assay. The radiolabeled peptide (50 µL, ~3.7 MBq) was then added to the test well. The control well contained phosphoramidon, a potent NEP inhibitor, at a final concentration of 1 mmol/L in addition to the contents of the test well. After 1 h incubation, all reactions were quenched using equal volume of CH_3_CN and centrifuged at 13,000 rpm for 10 min. The resulting supernatant was collected and analyzed using the analytical HPLC column to identify and quantify radio-metabolite(s). The assay was performed in duplicates.

### 4.7. Cell Culture

Human prostate cancer LNCaP cells obtained from ATCC (Manassas, VA, USA) were cultured in RPMI 1640 medium, supplemented with 10% FBS, penicillin (100 U/mL), and streptomycin (100 μg/mL) at 37 °C in a Panasonic Healthcare (Tokyo, Japan) MCO-19AIC humidified incubator containing 5% CO_2_. At 80−90% confluency, cells were washed with sterile phosphate-buffered saline, trypsinized and pooled before they were manually counted using a Bal Supply (Sylvania, OH, USA) 202C counter.

### 4.8. PET/CT Imaging and Ex Vivo Biodistribution in Tumor-Bearing Mice

All imaging and biodistribution studies were performed using male NOD-*scid* IL2Rg^null^ (NSG) mice and conducted according to the guidelines established by the Canadian Council on Animal Care and approved by Animal Ethics Committee of the University of British Columbia. For tumor inoculations, mice were anesthetized by inhalation with 2% isoflurane in oxygen and implanted subcutaneously with 5 × 10^6^ LNCaP cells below the left shoulder. Imaging and biodistribution studies were performed only after tumors grew to 5−8 mm in diameter over a period of 5−7 weeks.

For PET/CT imaging studies, ~3−6 MBq of the ^68^Ga-labeled tracer was injected through the tail vein. For the blocking study, 2-PMPA (0.2 mg) was co-injected with the tracer. Mice were allowed to recover and roam freely in the cages after injecting the tracer. At 45 min p.i., mice were sedated again and positioned on the scanner. First, a 10 min CT scan was conducted for localization and attenuation correction for reconstruction of PET images, before a 10 min PET image was acquired. Heating pads were used during the entire procedure to keep the mice warm.

For ex vivo biodistribution studies, mice were injected with ~1.5−3 MBq of the ^68^Ga-labeled tracer. At 1 h p.i., mice were euthanized, blood was drawn from heart, and organs/tissues of interest were collected, rinsed with PBS, blotted dry, weighed, and counted using an automated gamma counter. The uptake in each organ/tissue was normalized to the injected dose and expressed as the percentage of the injected dose per gram of tissue (%ID/g).

### 4.9. Quantification of Radio-Metabolites in Blood and Urine

Male NSG mice were injected with 3–17 MBq of the ^68^Ga-labeled peptide. For blood profiling, mice were anesthetized with 2% isoflurane in O_2_ and euthanized by CO_2_ inhalation at 5 min p.i. Blood draw was then performed by cardiac puncture and blood was collected in an eppendorf tube with equal volume of CH_3_CN. Each tube was then centrifuged at RT for 10–15 min and the resulting supernatant was collected and analyzed using the analytical HPLC column to identify and quantify radio-metabolite(s) in blood. For the purpose of urine profiling, urine was collected after euthanizing the mice at 15 min p.i. The urine samples were also collected and analyzed using the analytical HPLC column to identify and quantify radio-metabolite(s) in urine.

### 4.10. Statistical Analysis

Statistical analyses were performed by Student’s *t*-test using the Microsoft (Redmond, WA, USA) Excel software. The unpaired, two-tailed test was used to compare tissue uptake and tumor-to-background (muscle, blood and kidney) contrast ratios of [^68^Ga]Ga-DOTA-AmBz-MVK(HTK01166)-OH and [^68^Ga]Ga-DOTA-AmBz-M(O)VK(HTK01166)-OH. The unpaired, one-tailed test was used to compare kidney uptake of [^68^Ga]Ga-DOTA-AmBz-MVK(HTK01166)-OH (or [^68^Ga]Ga-DOTA-AmBz-M(O)VK(HTK01166)-OH) with that of the previously reported [^68^Ga]Ga-HTK01166 [15]. The difference was considered statistically significant when the *p* value was <0.05.

## Figures and Tables

**Table 1:** Biodistribution data and uptake ratios of ^68^Ga-labeled PSMA-targeted tracers in LNCaP tumor-bearing mice acquired at 1 h p.i. (*** *p* < 0.001).

Tissue (%ID/g)	[^68^Ga]Ga-DOTA-AmBz- MVK(HTK01166)-OH (n = 6)	[^68^Ga]Ga-DOTA-AmBz- M(O)VK(HTK01166)-OH (n = 6)
Blood	0.55 ± 0.09	0.56 ± 0.13
Urine	187 ± 53.8	221 ± 56.2
Fat	0.72 ± 0.33	0.52 ± 0.13
Testes	0.36 ± 0.06	0.31 ± 0.07
Intestines	0.40 ± 0.10	0.29 ± 0.10
Stomach	0.11 ± 0.02	0.13 ± 0.05
Pancreas	0.60 ± 0.22	0.56 ± 0.24
Spleen	8.87 ± 2.63	11.48 ± 2.70
Adrenal Glands	5.59 ± 4.02	4.33 ± 2.07
Kidneys	104 ± 12.2	89.7 ± 19.3
Liver	0.58 ± 0.08	0.32 ± 0.06 ***
Heart	0.25 ± 0.12	0.23 ± 0.05
Lungs	0.96 ± 0.12	0.88 ± 0.10
LNCaP Tumor	3.98 ± 0.87	4.06 ± 1.27
Muscle	0.21 ± 0.05	0.22 ± 0.05
Bone	0.16 ± 0.04	0.11 ± 0.05
Brain	0.02 ± 0.00	0.03 ± 0.01
Tail	1.03 ± 0.82	1.86 ± 2.44
Tumor/Muscle	19.9 ± 5.41	17.9 ± 7.57
Tumor/Blood	7.24 ± 1.16	6.83 ± 2.81
Tumor/Kidney	0.04 ± 0.01	0.04 ± 0.02

## Supplementary Materials

The following are available online at https://www.mdpi.com/1420-3049/25/17/3854/s1, Table S1: HPLC conditions and retention times for the purification of DOTA-conjugated peptides and peptidomimetics using the semipreparative column (Luna C18, 5 µm particle size, 100 Å pore size, 250 × 10 mm), Table S2: HPLC conditions and retention times for the purification of nonradioactive Ga-complexed DOTA-conjugated peptides and peptidomimetics using the semipreparative column (Luna C18, 5 µm particle size, 100 Å pore size, 250 × 10 mm), and Table S3: HPLC conditions and retention times for the purification/QC of 68Ga-labeled DOTA-conjugated peptides and peptidomimetics using the semipreparative column—Luna C18, 5 µm particle size, 100 Å pore size, 250 × 10 mm; the analytical (QC) column—Luna C18, 5 µm particle size, 100 Å pore size, 250 × 4.6 mm.
