# Supplementary material for: Evaluation of Met-Val-Lys as a Renal Brush Border Enzyme-Cleavable Linker to Reduce Kidney Uptake of 68Ga-Labeled DOTA-Conjugated Peptides and Peptidomimetics
Source: Molecules. 2020 Aug 25;25(17):3854. doi: 10.3390/molecules25173854 (PMC7503470; doi:10.3390/molecules25173854)
Supplement: Supplementary file 1 [file molecules-25-03854-s001.pdf]

Supplemental Table 1: HPLC conditions and retention times for the purification of DOTA-conjugated peptides and peptidomimetics using the semipreparative column (Luna C18, 5 μm particle size, 100 Å pore size, 250 x 10 mm).

Ligand	HPLC conditions	Retention time (min)
DOTA-AmBz-Met-OH	17% CH_3CN and 0.1% TFA in H_2O; flow rate: 4.5 min	10.2
DOTA-AmBz-MVK(Ac)-OH	16% CH_3CN and 0.1% TFA in H_2O; flow rate: 4.5 min	14.1
DOTA-AmBz-MVK(HTK01166)-OH	27% CH_3CN and 0.1% TFA in H_2O; flow rate: 4.5 min	12.6
DOTA-AmBz-M(O)VK(HTK01166)-OH	26% CH_3CN and 0.1% TFA in H_2O; flow rate: 4.5 min	8.3

Supplemental Table 2: HPLC conditions and retention times for the purification of nonradioactive Ga-complexed DOTA-conjugated peptides and peptidomimetics using the semipreparative column (Luna C18, 5 μm particle size, 100 Å pore size, 250 x 10 mm).

Ligand	HPLC conditions	Retention time (min)
Ga-DOTA-AmBz-Met-OH	17% CH_3CN and 0.1% TFA in H_2O; flow rate: 4.5 min	10.5
Ga-DOTA-AmBz-MVK(Ac)-OH	18% CH_3CN and 0.1% TFA in H_2O; flow rate: 4.5 min	11.2
Ga-DOTA-AmBz-MVK(HTK01166)-OH	26% CH_3CN and 0.1% TFA in H_2O; flow rate: 4.5 min	8.0
Ga-DOTA-AmBz-M(O)VK(HTK01166)-OH	26% CH_3CN and 0.1% TFA in H_2O; flow rate: 4.5 min	10.8

Supplemental Table 3: HPLC conditions and retention times for the purification/QC of ^{68}Ga-labeled DOTA-conjugated peptides and peptidomimetics using the semipreparative column - Luna C18, 5 μm particle size, 100 Å pore size, 250 x 10 mm; the analytical (QC) column - Luna C18, 5 μm particle size, 100 Å pore size, 250 x 4.6 mm.

Ligand	HPLC conditions		Retention time (min)
[^{68}Ga]Ga-DOTA-AmBz-MVK(Ac)-OH	Semi-prep	16% CH_3CN and 0.1% TFA in H_2O; flow rate: 4.5 min	24.3
	QC	18% CH_3CN and 0.1% TFA in H_2O; flow rate: 2.0 min	7.0
[^{68}Ga]Ga-DOTA-AmBz-MVK(HTK01166)-OH	Semi-prep	26% CH_3CN and 0.1% TFA in H_2O; flow rate: 4.5 min	26.1
	QC	28% CH_3CN and 0.1% TFA in H_2O; flow rate: 2.0 min	8.2
[^{68}Ga]Ga-DOTA-AmBz-M(O)VK(HTK01166)-OH	Semi-prep	23% CH_3CN and 0.1% COOH in H_2O; flow rate: 4.5 min	15.7
	QC	24% CH_3CN and 0.1% COOH in H_2O; flow rate: 2.0 min	6.2
